# Neutrophil Extracellular Traps Affecting Cardiovascular Health in Infectious and Inflammatory Diseases

**DOI:** 10.3390/cells10071689

**Published:** 2021-07-04

**Authors:** Manovriti Thakur, Bryce Evans, Marc Schindewolf, Iris Baumgartner, Yvonne Döring

**Affiliations:** 1 Division of Angiology, Swiss Cardiovascular Center, Inselspital, Bern University Hospital, University of Bern, CH-3010 Bern, Switzerland; manovriti.thakur@insel.ch (M.T.); bryce.evans@insel.ch (B.E.); marc.schindewolf@insel.ch (M.S.); iris.baumgartner@insel.ch (I.B.); 2 Institute for Cardiovascular Prevention (IPEK), Ludwig-Maximilians-University Munich (LMU), 80336 Munich, Germany; 3 DZHK (German Centre for Cardiovascular Research), Partner Site Munich Heart Alliance, 80336 Munich, Germany

**Keywords:** CVD, NETs, infection, inflammation, atherosclerosis, thrombosis, autoimmune diseases

## Abstract

Neutrophil extracellular traps (NETs) are web-like structures of decondensed extracellular chromatin fibers and neutrophil granule proteins released by neutrophils. NETs participate in host immune defense by entrapping pathogens. They are pro-inflammatory in function, and they act as an initiator of vascular coagulopathies by providing a platform for the attachment of various coagulatory proteins. NETs are diverse in their ability to alter physiological and pathological processes including infection and inflammation. In this review, we will summarize recent findings on the role of NETs in bacterial/viral infections associated with vascular inflammation, thrombosis, atherosclerosis and autoimmune disorders. Understanding the complex role of NETs in bridging infection and chronic inflammation as well as discussing important questions related to their contribution to pathologies outlined above may pave the way for future research on therapeutic targeting of NETs applicable to specific infections and inflammatory disorders.

## 1. Introduction

Neutrophils play a key role in the body’s innate immune response constituting the first line of defense against a wide range of pathogens. Neutrophils contribute to elimination of the intruder by phagocytosis, degranulation of antibacterial proteins, generation of reactive oxygen species (ROS) and the recruitment and activation of other immune cells. Moreover, neutrophils may form neutrophil extracellular traps (NETs), which are strands of DNA extruded by activated or dying neutrophils, decorated with various inflammatory mediators and represent a link between infection, inflammation, innate immunity, thrombosis and cardiovascular disease (CVD) [1]. In response to a growing list of stimuli including various bacterial and viral specimens, but also sterile particles such as urate crystals, neutrophils undergo a specialized series of reactions resulting in chromatin decondensation and subsequent NETs formation. The DNA that comprises the backbone of these NETs not only provides a scaffold for proteins stemming from the neutrophil itself but also for external proteins from other sources. Examples of neutrophil-derived proteins associated with NETs include myeloperoxidase, cathepsin G, neutrophil elastase (NE) and proteinase 3. A non-neutrophil protein associated with NETs would be, e.g., tissue factor (TF) a main trigger of the coagulation cascade fostering thrombosis [2]. Although NETs play a beneficial role in host defense against pathogens, there is increasing evidence that the presence of NETs (also in chronic inflammatory conditions) triggers a cascade of unwanted side effects, such as production of autoantibodies against the host’s DNA, tissue damage or induction of atherothrombotic events. In this review we will summarize recent findings of how NETs formation initiated by bacterial or viral infections as well as in autoimmune diseases contributes to complications of CVD. Uncontrolled NETs generation during bacterial and viral infections could be the missing link between infections and increased CVD risk [3].

## 2. NETs Affecting Cardiovascular Health in Infectious Diseases

In 2004, Brinkmann and colleagues showed the association of NETs with both Gram-positive and Gram-negative bacteria and showed bactericidal properties [4]. Since then, NETs have emerged as important players in host defense against various bacterial and viral infections. During infections, NETs induce entrapment, reduce dissemination and help in killing microorganisms. Failure of NETs-induced capturing and killing of microorganisms is associated with sepsis. NETs are prevalent during sepsis, as they control the spread of infection, and their degradation, using recombinant DNase, leads to increased bacterial burden (shown in mice) but at the same time leads to organ and tissue damage [5]. In a similar study, NETs were associated with platelet aggregates, triggering microvessel occlusion in septic mice [6]. In addition to this, NETs-induced hepatic damage was observed in methicillin-resistant *S. aureus* induced sepsis. This damage was inhibited by NE or peptidyl arginine deiminase type IV (PAD4) deficiency [7]. Similarly, inhibition of NETs using DNase1 or neutralizing antibodies against histones tend to reduce organ damage in a poly-microbial sepsis mouse model [8]. Acute infection with various viruses and Gram-positive and Gram-negative bacteria leads to higher risk of CVD [9,10]. In this section, we will focus on role of NETs in major bacterial and viral infections leading to cardiovascular complications. The inducers of NETs formation during infection and their role in CVD manifestation have been summarized in Table 1.

### 2.1. Cardiovascular Manifestations of Bacterial NETs Induction

*Staphylococcus aureus* is a Gram-positive bacterium that causes diseases such as osteomyelitis, infective endocarditis, bacteremia and gastroenteritis by inducing NETs formation and severe inflammation [4,43]. *S. aureus* leads to conversion of NETs to deoxyadenosine that exerts caspase-3-mediated cytotoxic effects on macrophages. This mechanism helps *S. aureus* to escape NETs induced entrapment [44]. Infection with methicillin-resistant *S. aureus* (MRSA) was shown to induce NETs formation in liver vasculature, which leads to liver damage [7]. NETs entrap bacterial-platelet aggregates and promote septic thrombus formation on injured heart valves, as shown in an *S. aureus*-induced endocarditis rat model. This suggests a role of NETs in bacterial biofilm production and platelet aggregation on injured heart valves [14]. In the same study, inhibition of NETs formation by administration of DNase1 before infecting the rats with *S. aureus* leads to reduction in biofilm and septic thrombus formation [14]. Another Gram-positive bacterium, *Streptococcus pneumonia*, also leads to induction of NETs formation [4]. A pneumococcal protein called α-enolase, from *S. pneumonia*, interacts with neutrophil surface proteins, increases neutrophil recruitment and induces NETs formation [11]. In a model of invasive pneumococcal disease in mice, *S. pneumonia* was shown to invade the myocardium and lead to formation of microlesions. These microlesions in the invaded myocardium also display high neutrophil and macrophage infiltration. Ongoing accumulation of *S. pneumonia* in these microlesions induces cardiomyocyte cell death within the lesion [45].

*Escherichia coli* is a Gram-negative bacterium causing pathologies such as enteritis, urinary infections, meningitis and sepsis [46]. Serum from patients with *E.coli*-triggered septic shocks leads to significant NET formation by neutrophils in vitro [47]. Exposure to *E.coli*-derived lipopolysaccharides (LPS) activates the coagulation system through individual histone proteins and human neutrophil DNA [48]. LPS-stimulated platelets are shown to promote NETs formation through Toll-like receptor 4 (TLR4) activity [12], and platelet, thrombin and NETs interactions trigger intravascular thrombus formation in the liver microcirculation of septic mice [17]. In the same study, PAD4-deficient animals, which have a reduced ability to form NETs, showed a significant decrease in microvascular thrombosis [17]. Another study highlighted the link between acute infection, NETs and vascular inflammation by using a mouse model of endotoxemia. Here, they induced endotoxemia by injecting LPS to *Apoe^−/−^* mice fed with a Western diet (WD) for 4 weeks, which resulted in accumulation of NETs along the arterial wall, recruitment of myeloid cells to the atherosclerotic lesions and augmented lesion size [16]. Furthermore, the authors showed that the interaction of NETs and monocytes was not regulated by a receptor but instead by cationic histone H2a. Cationic histone H2a electrostatically interacts with the negatively charged monocyte surface and leads to enhanced monocyte adhesion to the arterial wall and increased recruitment to the atherosclerotic plaque. In the same study, inhibition of NETs by Cl-amidine, a second-generation PAD inhibitor replacing the phenyl and carboxamide groups of Cl-amidine with a biphenyl and benzimidazole group, respectively, significantly reduced cell-free DNA in the plasma, eliminated the NETs in the lumen and inhibited myeloid cell recruitment to the atherosclerotic lesion [16].

*Mycobacterium tuberculosis* specifically affects the respiratory system, causing tuberculosis. Different subspecies of mycobacterium can induce NETs when they are cultured with neutrophils, for instance, *M. tuberculosis* induced NETs formation by secreting early secretory antigen-6 protein resulting in elevated intracellular Ca^2+^ in neutrophils and causing necrosis [13]. NETs can effectively trap and reduce the dissemination of *M. tuberculosis*; however, NETs-derived components are unable to kill *M. tuberculosis* [49]. Latent tuberculosis infection is associated with acute myocardial infarction and may trigger cardiovascular complications [15]. In a case study, mass spectrometry analyses of the composition of infective endocarditis biofilm and septic thrombus showed the contribution of NETs to the formation of tuberculous endocardiac mass formation [18]. These tuberculosis endocardiac masses are space-occupying lesions in the endocardium consisting of bacterium, platelets, fibrin, microorganisms and inflammatory cells caused by endovascular microbial infection of intracardiac structures facing the blood. Endocardiac mass formation can lead to endcocardial damage and valve destruction. In one case study where a patient presented with a large mass on the anterior mitral leaflet and dense yellow areas inside the right atrium, these areas were positive for *M. tuberculosis* and were sensitive to all anti-tuberculous medications [50]. Patients with tuberculosis endocarditis were managed with valve replacement and anti-tuberculosis drugs [51]. Altogether, bacterial infections promote progression of atherothrombosis or atherosclerosis possibly through induction of NETs formation, subsequent formation of platelet–NETs aggregates and NETs-induced cardiac damage or fostering of atherosclerotic lesion growth.

### 2.2. Cardiovascular Manifestations of Viral NET Induction

Many viruses such as *Hemagglutinin Type 1 and Neuraminidase Type 1* (H1N1), Human Immunodeficiency Virus (HIV) and hepatitis have either a direct or an indirect effect on CVD. The mechanisms behind these effects are still not fully understood. In addition, NETs involvement in CVD during viral infections has received little attention. This oversight may be due to the fact that many viruses have developed many strategies to prevent the formation of NETs or escape them. Therefore, they have not been investigated in the context of viral co-morbidities such as CVD. For example, Hepatitis B virus may inhibit NETs release by modulating reactive oxygen species production and autophagy [52]. HIV has developed many mechanisms to evade the human immune system and can even induce neutropenia by impairing hematopoiesis [53]. HIV also choreographs abnormal neutrophil behavior including chemotaxis, phagocytosis, oxidative metabolism and pathogen-killing [54]. Neutrophils from HIV-infected individuals exhibit elevated interleukin 12 (IL-12) secretion triggering a chronic inflammatory process [55]. As a counter measure, HIV induces the release of the anti-inflammatory IL-10 from dendritic cells to neutralize NETs [56]. Human IL-10 homologs have been found in the genome of DNA viruses such as human cytomegalovirus (HCMV) and Epstein–Barr virus (EBV) [57,58]. These IL-10 homologs could suppress NETs formation in a similar manner to cellular IL-10. Like cellular IL-10 these homologs inhibit the TLR 7 and TLR 8-signaling pathways, thus suppressing NADPH oxidase-dependent formation of NETs [56,59]. Since the 1930s a correlation between influenza infections and vascular complications has been observed [60,61,62]. A recent study also highlighted that excessive NETs formation, neutrophil inflammation and delayed apoptosis is found in patients with severe influenza infection [63]. These findings suggest that neutrophils may dominate the host response towards influenza, and this correlates with poorer outcomes. Therefore, excessive NETs formation could also have downstream implications such as the development of CVD. Atherosclerosis-prone *apolipoprotein E-deficient* (*Apoe^−/−^*) mice are a popular model for the study of atherosclerosis as they have a reduced lipoprotein clearance and accumulation of cholesterol ester-enriched particles in the blood promoting the development of atherosclerotic plaques [64]. A study has demonstrated that *Apoe^−/−^* mice infected with the influenza A virus, show direct infection of vascular endothelial cells and smooth muscle cells in atherosclerotic plaques [65]. This study further showed that influenza infection promotes inflammation, smooth muscle cell proliferation and fibrin deposition in atherosclerotic plaques. *Apoe^−/−^* mice infected with influenza A showed increased cell infiltration in atherosclerotic plaques compared to uninfected *Apoe^−/−^* mice. However, wild-type infected C57BL/6 mice showed no evidence of the same infiltration of the vascular intima [65]. This could suggest that in patients with atherosclerosis, influenza A infections could exacerbate the disease. There is already some evidence proposing that the influenza vaccine can reduce the risk of developing CVD [66]. Another study demonstrated that human leukocytes adhere to influenza-infected human umbilical vein endothelial cells (HUVECs) in a viral dose-dependent manner [67]. Further investigations on human aortic endothelial cells (HAECs) revealed that influenza virus infections increase the expression of chemokines, including chemokine (C-C motif) ligand 2 (CCL2), CCL5, and IL-8. Furthermore, influenza infection augmented the expression of intercellular adhesion molecule 1 (ICAM1), vascular cell adhesion molecule 1 (VCAM-1) and E-selectin on human aortic endothelial cells (HAECs) and human umbilical vein endothelial cells (HUVECs) [68,69]. Together, these data suggest that the influenza virus may exacerbate atherosclerosis, as it enhances the pro-inflammatory environment and leukocyte infiltration. Therefore, this increase in inflammation in the vasculature would likely result in the infiltration of neutrophils and possibly the formation of NETs. The cytotoxic effect of NETs could create endothelial dysfunction setting the stage for the development of atherosclerosis. However, this would require further investigation.

Influenza A WSN/33(H1N1) virus can also induce host cell proteases, such as trypsin and matrix metalloprotease 9 (MMP-9), in various organs, which may act to increase vascular permeability and viral entry in different organs [70]. As of now, the role of neutrophils in atherosclerosis during influenza infections has not been investigated. NETs have cytotoxic effects on endothelial cells [71] and damage the lungs in mice infected with influenza A virus H1N1 strain PR8 [72]. Furthermore, NETs are found in the alveolar epithelium and in small blood vessels in areas of hemorrhagic lesions in the lung, probably causing alveoli-capillary damage [72]. A study has demonstrated that patients with severe influenza presented with elevated NETs in the plasma, and the neutrophils from these patients released a greater amount of myeloperoxidase-DNA (MPO-DNA) complex in response to IL-8 [73]. Furthermore, NETs from influenza A infections in H1N1 and H7N9 patients increased the permeability of alveolar epithelial cells [73]. It is conceivable that influenza infections could trigger NETs in the circulatory system and either induce atherosclerosis due to increasing permeability of vascular endothelial cells or exacerbate already established atherosclerosis plaques. However, further research is essential to understand the role of NETs in CVD during influenza infections.

In 2019 a novel virus, *severe acute respiratory syndrome coronavirus type 2 (SARS-CoV-2)*, was identified which caused an atypical respiratory disease. It is a multifaceted disease affecting the circulatory system as well as the respiratory system. Increased neutrophil counts in COVID-19 correlate with disease severity, and poor prognosis and extensive neutrophil infiltration of pulmonary capillaries has been observed in autopsy studies [74,75]. The angiotensin-converting enzyme 2 (ACE2) has been identified as one of the most important receptors for the virus entry into host target cells. During hypoxia, angiotensin II induces pulmonary vasoconstriction to restore the ventilation–perfusion imbalance, while simultaneously inducing adverse pro-fibrotic effects. However, both vasoconstriction and fibrosis are associated with upregulation of ACE2 [76,77]. The upregulation of ACE2 in patients with comorbidities may result in an increased viral load and spreading of infection to extrapulmonary tissues [19]. This systemic infection is associated with higher neutrophil-to-lymphocyte ratios in infected tissues and high levels of pro-inflammatory cytokines [78,79]. Evidence suggests that microthrombi and coagulopathy contribute to COVID-19 pathogenesis [78,79]. It has been demonstrated that COVID-19 increases the levels of intracellular reactive oxygen species (ROS) in neutrophils to stimulate NETs formation [19]. Another study has demonstrated that during COVID-19 infections, neutrophils are alternatively activated, express more CD64 and programmed death-ligand 1 (PD-L1) [80] and could be the reason for excessive NETs formation in COVID-19 [21]. Additional evidence has suggested that neutrophils in severe COVID-19, guided by activated platelets, migrate into inflamed tissue and release NETs [81,82,83]. A recent study showed microvascular thrombi associated with platelet–neutrophil aggregates in the lung, kidney and the heart of COVID-19 patients [22]. In a cohort study, plasma MPO-DNA complexes defined as NETs, platelet factor 4, RANTES (CCL5) and selected cytokines were measured in plasma samples from COVID-19 patients (*n* = 33) and age- and sex-matched controls (*n* = 17) [83]. This study revealed that an increased prevalence of MPO-DNA complexes in COVID-19 patients correlated directly with the severity of the disease. Furthermore, three COVID-19 lung autopsies were analyzed for NETs and platelet involvement, revealing NETs-containing microthrombi with neutrophil-platelet infiltration within these samples [83]. The latter suggests that NETs are not only involved in the host defense against SARS-CoV-2 but may also trigger detrimental immunothrombosis, a process understood as the interaction of activated leukocytes with platelets and plasma coagulation factors resulting in thrombotic complications of COVID-19 patients [21,84]. Others investigated how complement interacts with the platelet-induced-NETs–thrombin axis during COVID-19 and found increased plasma levels of NETs, tissue factor (TF) activity and sC5b-9 in COVID-19 patients [85]. Neutrophils isolated from COVID-19 patients had higher TF expression and released NETs carrying active TF. Treatment of control neutrophils with COVID-19 platelet-rich plasma generated TF-bearing NETs that induced endothelial dysfunction. The inhibition of thrombin, NETs formation or C5aR1 blockade resulted in the attenuation of platelet-mediated NETs-driven thrombogenicity [85]. COVID-19 serum further induced complement activation in vitro, consistent with high complement activity found in clinical samples, and the inhibition of complement disrupted TF expression in neutrophils [85]. Overall, these data demonstrate that complement and NETs may play an important role in COVID-19-associated immunothrombosis. Patients hospitalized with COVID-19 exhibited elevated levels of neutrophil activation and NETs formation, which are associated with higher risk of death due to thrombotic complications in these patients [86].

These observations highlight the urgent need for further research on the potential relationship between NETs-induced thrombosis in COVID-19. As the level of neutrophils and NETs in patients with COVID-19 has a direct correlation with severity, therapeutics targeting NETs could improve the outcome [87]. Hopefully this will encourage future research to investigate the role of NETs in other viral infections and their influence on CVD.

## 3. Implications of NETs Formation in CVD

**Figure 1:**
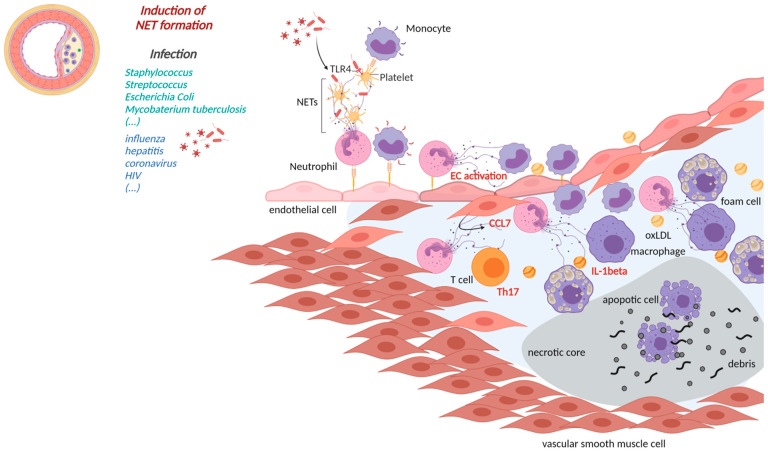
NETs in atherosclerosis. (**Left**): cross section of an atherosclerotic vessel; (**Middle**): pathogens associated with the induction of NETs formation; (**Right**): Close up atherosclerotic lesion; Neutrophils activated e.g., by bacterial and viral pathogens may undergo NETs formation thereby activating leukocytes, platelets and endothelial cells creating a pro-inflammatory milieu triggering lesion growth. Lesional NETs may be induced by pathogens or CCL7 and initiate a IL-1beta release or a Th17 response altogether driving lesion expansion. (made with biorender.com, 4 June 2001).

**Figure 2:**
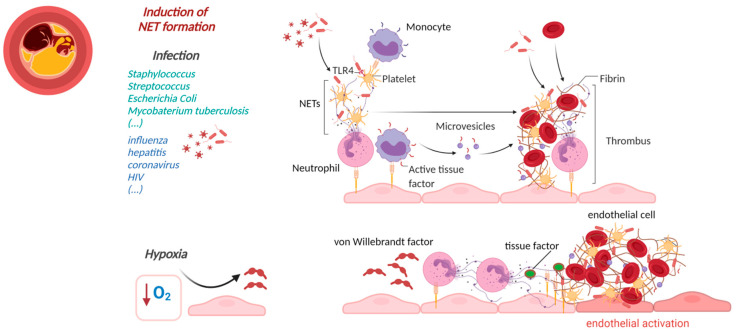
NETs-initiated thrombosis. (**Left**): Cross section of a thrombus occluded artery. (**Middle**): NETs initiators; (**Right**): Pathogens may activate neutrophils to form neutrophil extracellular traps (NETs) which support (immuno)thrombosis e.g., through histone-dependent platelet activation or binding to von Willebrand factor (VWF) and display of tissue factor within the NETs structure. (made with biorender.com, accessed on 4 June 2001).

In recent years, NETs emerged as key players in the progression of CVD. Aging, being one of the major risk factors for CVD, is also associated with a higher susceptibility of neutrophils to form NETs, cardiac fibrosis, heart failure and atherosclerosis. In an experimental model of cardiac fibrosis, higher NETs formation was observed in older C57BL/6J mice (20–27 months) as compared to younger mice of 8–16 weeks of age [88]. In the same study, PAD4 deficiency or DNase1 treatment (to inhibit NETs) resulted in diminished platelet–neutrophil complexes and reduced fibrosis in the myocardium of older mice [88]. Mitochondrial oxidative stress in myeloid cells in aged mice (36 weeks old) led to enhanced NETs in the atherosclerotic plaque and contributed to enhanced atherosclerosis [89]. Presence of NETs in atherosclerotic plaques implies an important role of NETs in atherosclerosis [90]. Analyses of blood samples drawn from patients before and after percutaneous coronary intervention (PCI) suggested high levels of NETs markers such as double-stranded (ds)DNA and MPO-DNA after PCI. Higher circulatory levels of dsDNA at day 1 after PCI were associated with large infarct size, and their prevalence indicates adverse cardiovascular complications [91]. However, dsDNA could be associated with other cell types, and further NETs-specific investigations will be required. In another study, NETs and NET components are shown to have pro-thrombotic properties as they promote coagulation by inducing fibrin network formation [92]. Therefore, in this section, we will focus on their induction, role in vascular manifestation (Table 1) and involvement in atherosclerosis (Figure 1) and vascular thrombosis (Figure 2).

### 3.1. NETs in Atherosclerosis

Atherosclerosis is a chronic inflammation of the arterial wall, initiated through luminal endothelial cell activation and damage, intimal accumulation of low-density lipoproteins (LDLs) and recruitment of monocytes, dendritic cells, neutrophils and T-lymphocytes to the intimal layer. Infiltrated monocytes mature into macrophages and eventually lead to foam cell formation, thus leading to progression of atherosclerotic plaque formation [93].

High neutrophil numbers in atherosclerotic plaques in the coronary arteries was reported to cause plaque erosion and rupture [94]. Another study focusing on monocyte-depleted *Lysm^egfp/egfp^ Apoe^−/−^* mice after four weeks of WD and on human atherosclerotic plaques obtained by endarterectomy have reported the presence of NETs in mouse and human atherosclerotic plaques [90]. Another study showed the differential presence of NETs in upstream and downstream regions of human samples isolated during carotid endarterectomy. They further established that patients with high serum levels of autoantibodies against apolipoprotein A-1 (ApoA1) presented with a higher signal for citrullinated histone H3 outside of neutrophils when compared to serum negative patients. This indicates that expression of NETs elements could be influenced by circulating levels of anti-ApoA-1 [26]. NETs formation can also occur in early stages of atherosclerosis induced by cholesterol crystals leading to higher transcription of pro-inflammatory mediators such as IL-1β and IL-6 from primed lesional macrophages [23]. IL-1β and IL-6 can further activate T helper cell 17 (Th17) cells and lead to accelerated recruitment of immune cells into the atherosclerotic plaque (Figure 1) [23].

Analyses of smooth muscle cell rich fibrous caps in mouse and human atherosclerotic plaques showed the presence of NETs releasing neutrophils. Furthermore, the supernatant of activated smooth muscle cells attracts neutrophils via CCL7 and stimulates NETs formation. This NETs induction resulted in histone H4 release, which leads to smooth muscle cell lysis and eventually participates in a vulnerable plaque phenotype. Neutralization of histone H4 in hypercholesterolemic mice resulted in enhanced lesional smooth muscle cell content and generated reduced vulnerability in the plaques [29]. This underlines the role of NETs in inducing vulnerable plaques, which could result in plaque rupture-induced thrombotic complications (Table 1). Patients with atherosclerosis show high serum levels of IL-8, a pro-inflammatory cytokine, and NETs in blood. IL-8 interacts with the CXC chemokine receptor 2 (CXCR2) on neutrophils and induces NETs formation. CXCR2 depletion by using CXCR2 blocking antibody inhibited NETs formation and alleviated atherosclerosis progression in *Apoe^−/−^* mice fed with WD for 12 weeks [24]. In another study, exosomes extracted from oxidized LDL treated HUVECs resulted in *metastasis-associated lung adenocarcinoma transcript 1* (MALAT1)-induced NETs formation and atherosclerosis progression [27]. Additionally, modified lipids such as oxidized LDL co-incubated with HL-60-derived neutrophils and human peripheral blood neutrophils lead to significantly higher NETs formation [25]. In turn, NETs formation can induce damage to endothelial cells because in smooth muscle cell rich plaques apoptotic endothelial cells were correlated with more NETs-rich regions (Table 1) [28]. Cathepsin G (one of the components of NETs) and cathelicidin-related antimicrobial peptides can induce activation of monocytes and dendritic cells, fostering monocyte adhesion to endothelial cells and eventually influencing their recruitment to the atherosclerotic lesions [95,96]. NETs can also stimulate macrophages to produce pro-inflammatory cytokines, which result in Th 17 cell activation and a significant increase in immune cell recruitment to the atherosclerotic plaque [97]. Moreover, repetitive social defeat exposure in *Apoe^−/−^* mice promoted atherosclerosis by promoting NETs formation within the plaque. DNase I treatment to inhibit NETs in these animals resulted in reduction of pro-atherogenic effects of repetitive social defeat exposure [98]. NETs in atherosclerotic plaques are associated with a pro-inflammatory phenotype of macrophages (Table 1). Resolution of NETs by injecting DNase 1 to male *Ldlr^–/–^* mice fed with WD for 16 weeks reduced NETs-induced plaque macrophage inflammation and suppressed atherosclerosis progression in diabetic mice [31]. Another study suggests that inflammasome activation could lead to increased neutrophil recruitment and NETs formation in atherosclerotic plaques. Cholesterol transporters *ATP Binding Cassette* (ABC) A1 and G1 promote cholesterol efflux to high-density-lipoprotein (HDL), and *Abca1/g1* deficiency in myeloid cells leads to cholesterol accumulation. Cholesterol accumulation in myeloid cells activates the NLRP3 inflammasome, which enhances neutrophil accumulation and NETs formation in atherosclerotic plaques. NLRP3 deficiency shows to reduce atherosclerotic lesion size in myeloid *Abca1/g1*-deficient *Ldlr^−/−^* mice [30].

In a photochemical injury thrombosis model, *Apoe^−/−^* mice were treated for 11 weeks with Cl-amidine to block NETs formation, which resulted in reduced atherosclerotic lesion size and prolonged induction of carotid artery thrombosis in these animals [32]. Additionally, in the same study, combined administration of chloramidine and a neutrophil depletion antibody into *Apoe^−/−^* mice did not affect the lesion size. Another study used PAD4-deficient bone marrow cells to halt the ability of neutrophils to undergo NETs formation. This hematopoietic PAD4-deficiency was shown to inhibit intimal NETs formation in the carotid arteries of eight-week-old *Ldlr^−/−^* male mice fed with modified WD diet and led to protection of intimal integrity of the endothelium. Thrombus formation was also observed on the intimal surface in mice reconstituted with wild-type bone marrow as compared to those with *Pad4^−/−^* bone marrow [99]. In a similar study, myeloid-specific deletion of PAD4 substantially diminished NETs formation and NETs-induced atherosclerosis in the aorta of *Pad4^−/−^* mice fed with WD for 10 weeks [100]. Targeted delivery of GSK484, a PAD4 inhibitor, via collagen IV-targeted nanoparticles to reach lesions with features of superficial erosion, resulted in decreased NETs accumulation at the sites of intimal injury in 8-week-old *Apoe^−/−^* mice [101]. These findings point towards a crucial role of NETs and their inhibition in lesion size formation and thrombotic complications during atherosclerosis.

### 3.2. NETs-Induced Thrombosis

Numerous studies have indicated the presence of NETs in arterial and venous thrombi. NETs formation during thrombosis is thought to be initiated by hypoxia-induced release of von Willebrand factor (vWF) and P-selectin from the endothelium, which recruits and activates neutrophils that form NETs (Figure 2) [102,103,104]. NETs in return activate endothelial cells and induce VCAM-1 and ICAM-1 [102], thus providing a scaffold for adhesion of platelets, red blood cells, fibrinogen, vWF and fibronectin [92]. This scaffold not only forms a structural basis, but many of its components can also actively trigger platelet activation and blood coagulation. Furthermore, NETs also induce TF expression on the endothelial cell surfaces, thereby accelerating plasma clotting in vitro. Elevated TF expression in neutrophils and the release of NETs decorated with TF have been found not only in COVID-19 but also in patients with sepsis [47], ST-segment elevation acute myocardial infarction (STEMI) [105] and SLE [106]. This suggests that NETs activate the extrinsic pathway of coagulation, which is a shorter coagulation pathway of fibrin formation. It includes activation of coagulation factor VII to factor VIIa and thereby generation of factor Xa. Factor Xa further contributes to fibrin formation by activating prothrombin to thrombin with the help of Factor V as a cofactor [107].

Other component of NETs, such as histones H3 and H4, are highly cytotoxic to endothelial cells [108] and smooth muscle cells [29] and induce platelet aggregation via Toll-like receptor TLR 2 and 4 [92,108], which eventually promotes thrombin generation [92]. Histones further endorse thrombin generation by binding to thrombomodulin and prevent the activation of activated protein C (APC) [109]. NE and cathepsin G, which are also present on NETs, enhance TF- and factor XII-driven coagulation via proteolysis of tissue factor pathway inhibitor (TFPI) [110]. NETs also directly bind plasma factor XII and, with the cooperation of platelets, support its activation to plasma factor XIIa [111]. NETs can contribute towards fibrin formation through the intrinsic pathway of coagulation. NETs–microparticle complexes can also induce NETs-induced thrombin generation via the intrinsic pathway of coagulation since their incubation with factor XII-deficient plasma reduced NETs-induced thrombin generation by 97% [112]. Upon exposure to pathogens, NETs together with platelets and immune cells lead to the formation of intravascular thrombi that could entrap the pathogen and limit its dissemination (Figure 2). Intravascular NETs formation can lead to fibrin-independent immune cell-mediated occlusion of microvessels and cause massive cell death in affected areas [35,36]. Inhibitor of NETs, for instance, via injection of DNase 1 and DNase1-like 3, prevent vascular occlusion by degrading NETs clots during chronic neutrophilia [113]. In addition to this, patients with acute thrombotic microangiopathies also show low levels of plasma DNase 1 [114]. In line with this, N-deficient mice also show differences in thrombus formation, hence suggesting a role of NETs in vascular thrombosis [110].

In heparin-induced thrombocytopenia (HIT), which can clinically manifest in arterial and venous thromboembolic complications, heparin/platelet factor 4/IgG antibody complexes lead to platelet activation mediated through binding of platelets to FcγRIIa receptors, which consecutively propagates a massive auto-amplifying coagulation activation, by release of procoagulant factors. Recent data now also show that neutrophils can be directly activated by heparin/platelet factor 4/IgG antibody complexes through their FcγRIIa receptors, which also propagates NETs formation [115]. In line, the latter pathway cannot be inhibited by anti-CD62p and anti-CD162 antibodies to suppress NETs formation, but NETs generation and thrombus formation are abrogated in FcγRIIa+/heparin-platelet factor 4+ mice lacking PAD4 [115]. These results show that thrombosis in HIT, at least in part, is mediated through neutrophil activation and NETs induction.

Venous thromboembolism (VTE) is composed of deep vein thrombosis (DVT) and pulmonary embolism (PE). Various studies account for the presence of NETs in venous thrombi (Table 1). One study on iliac veins with balloon catheterization in baboons revealed an extracellular chromatin core in the thrombus. During thrombus formation, plasma levels of cell-free DNA were also elevated [92]. Similar findings were observed in an inferior vena cava DVT mouse model induced by flow restriction in mice [111,116]. In another study, 16 VTE thrombi originating from 11 patients with DVT, PE and/or an inferior vena cava filter thrombus were analyzed [117]. After scoring the organization stage based on the Masson trichrome staining, consecutive sections were analyzed for the presence of neutrophils and citrullinated histone H3 as a marker of extracellular NETs structures. These findings indicated that NETs formation takes place during recruitment of neutrophils to the thrombi and are replaced by collagen fibers at a later stage of thrombus formation.

NETs are also abundant in coronary thrombi, as observed and analyzed in patients with acute myocardial infarction [37,118]. In another study, investigating patients with stent thrombosis after percutaneous coronary intervention, neutrophils and NETs were abundantly found in the retrieved thrombi [119]. Analyses of NETs in coronary thrombi collected from ST-elevation acute coronary syndrome patients, via coronary thrombectomy, correlated positively with infarct size (Table 1) and negatively with ST-segment resolution [37]. Further, 68 ischemic stroke thrombi showed abundance of NETs [120]. Together, these findings point towards the potential clinical importance of NETs in arterial and venous thrombosis.

## 4. Cardiovascular Manifestations of Sterile NETs Induction

An autoimmune disease describes a pathophysiology where the host immune system attacks cells and or tissue of host origin (self) [121]. The disease can affect one or multiple parts of the body. Common symptoms include fever and tiredness, but autoimmune diseases can also be pro-inflammatory [121]. This pro-inflammatory environment induced by the disease can in turn trigger the development of NETs [122]. Key inducers of NETs formation and related vascular manifestations to enhance CVD risk are shown in Table 1. Systemic lupus erythematosus (SLE) is an autoimmune disease characterized by a loss of tolerance to self-antigens, abnormal T and B cell responses, autoantibody production and correlates with a higher risk of CVD, especially atherosclerosis [123]. A subset of immature neutrophils, called low-density granulocytes (LDGs), possessing a proinflammatory phenotype may contribute to disease pathogenesis [124]. NETs formation is also reported in SLE in vivo and was described in the kidney, skin and peripheral blood [38]. Thus, it is conceivable that NETs formation due to SLE, especially in the arteries, could activate the endothelium and initiate atherosclerotic lesion formation. Given that active neutrophils and NETs are in abundance in SLE patients, and that SLE patients have a higher risk of CVD, NETs could be an important link in this detrimental relationship.

Neutrophils have been described to have a multitude of irregularities in individuals with SLE. These neutrophils have impaired phagocytic potential [125,126] are unable to be cleared by the C1q/calreticulin/CD91-mediated apoptotic pathway [127] and show uncharacteristic oxidative activity [128,129]. Of note, SLE has a distinct granulocyte population of neutrophil LDGs [130], which promote the production of proinflammatory cytokines, including type I interferons (IFN-I), and cause endothelial cell damage (Table 1) [38,124]. These LDGs are capable of forming NETs, even in the absence of further stimulation, and are protected from degradation by nucleases [38,39,131]. NETs further promote plasmacytoid dendritic cells to increase production of IFN-α [39]. Moreover, they trigger the NLRP3 inflammasome in macrophages to enhance IL-1β and IL-18 production [131]. Together, this pro-inflammatory environment results in endothelial damage and dysfunction known to trigger atherosclerosis [38,42]. MMP-9 released by LDGs during NETs formation has been demonstrated to impair murine aortic endothelium-dependent vasorelaxation and induce endothelial cell apoptosis [40]. The inhibition of MMP-2 activation restored endothelial function and reduced NETs-induced vascular cytotoxicity [40]. This further highlights the capability of NETs in SLE to incite vascular endothelial dysfunction, which could result in the development of atherosclerosis. Another possible proatherogenic mechanism of NETs in SLE is the NETs-derived MPO/NADPH oxidase (NOX)/nitric oxide synthase (NOS)-generated oxidative species, which are elevated in SLE [42]. This could generate dysfunctional HDL in SLE as it may take place in the circulation rather than the vascular wall [132,133]. Due to its particle size, it is conceivable that HDL can be trapped by NETs in the blood vessel lumen, endothelium or sites of inflammation, rather than in subendothelial atherosclerotic lesions [90]. NETs-mediated entrapment of HDL may lead to a dysfunctional lipoprotein, impairing cholesterol efflux capacity and promoting proinflammatory responses in blood vessels. Taken together, these data suggest that NETs could either directly trigger or exacerbate atherosclerosis in SLE, as the formation of NETs in the vasculature could cause endothelial dysfunction and subsequent atherogenesis (Table 1) [41]. Alternatively, NETs can indirectly trigger or exacerbate by disruption of the cholesterol efflux by entrapping HLD and promoting proinflammatory responses in blood vessels, leading to atherosclerosis [41]. Furthermore, NETs in SLE could contribute to the formation of thrombi, as revealed in prothrombotic NZM2328 mice, which rapidly form carotid thrombi after photochemical injury of the endothelium. The latter was inhibited if NETs formation was blocked, either with DNase1 or a PAD inhibitor, and resulted in decreased formation of thrombi [134]. Thus, in autoimmune diseases where there is an excess of NETs formation, the risk of thrombus formation could be increased.

Whether or not infections, either viral or bacterial, in patients with an autoimmune disease such as SLE would have an effect on CDV is debatable. No study has investigated CVD in patients with autoimmune diseases during an infection. Hence, it is not clear if an infection would exacerbate the detrimental role NETs in a patient with an autoimmune disease and CVD. Infection could also change the phenotype of the NETs, reducing its impact on CVD or vice versa. Future work would be needed to better understand this phenomenon.

Rheumatoid arthritis (RA) is a chronic systemic disease characterized by joint inflammation and bone destruction. Evidence suggests that NETs are involved in the pathogenesis of RA [135]. For example enhanced NETs formation has been observed in circulating and synovial fluid in RA patients compared to healthy controls and from patients with osteoarthritis [135]. Neutrophils infiltrated RA synovial tissue, rheumatoid nodules and skin, and NETs abundance correlated with the presence of autoantibodies to citrullinated antigens (ACPA) and with systemic inflammatory markers [135]. CVD risk is increased in RA, but there is limited research on the involvement of NETs in atherosclerosis in patients with RA. A case–control study of patients with RA and new-onset coronary artery disease (CAD) (*n* = 75) was compared to an age-and sex-matched control group with newly diagnosed CAD (*n* = 128) [136]. This study demonstrated that patients with RA were more likely to have multivessel coronary involvement compared to the control group. There was no significant difference between the cohorts with regards to risk factors for CAD including diabetes, hypertension, hyperlipidemia and smoking history. However, until now it is not clear if NETs in lesions from RA patients differ from the ones found in atherosclerotic lesions and how RA NETs influence CVD and atherosclerosis.

Another study has demonstrated that self-DNA, possibly released during NETs formation, and an increased expression of the antimicrobial peptide Cramp/LL37 in atherosclerotic lesions may drive atherosclerosis [137]. Plasmacytoid dendritic cell depletion and cramp-deficiency in bone marrow of *Apoe^−/−^* mice resulted in the attenuation of atherosclerosis and anti-ds DNA antibody titers [137]. Thus, self-DNA and cramp could act to stimulate a plasmacytoid dendritic cell-driven pathway of autoimmune activation and the generation of anti–double-stranded-DNA antibodies to exacerbate atherosclerosis lesion formation.

**Figure 3:**
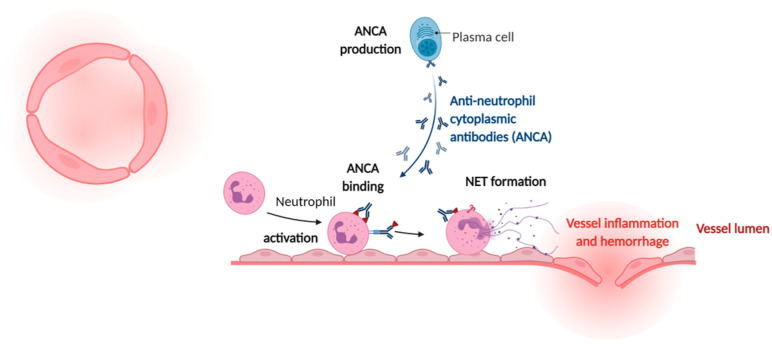
NETs in ANCA-associated vasculitis. (**Left**): Cross-section of a small vessel. (**Right**): Pathogenesis of ANCA-associated vasculitis is multifactorial, involving numerous immune cells. B lymphocytes feature prominently as producers of ANCAs, while activated neutrophils undergo NETs formation driving tissue damage via injury to the vascular endothelium (made with biorender.com, accessed on 4 June 2001).

Anti-neutrophil cytoplasmic antibody (ANCA)-associated vasculitis (AAV) is used to describe group of autoimmune diseases characterized by destruction and inflammation of small vessels. The presence of NETs was recently demonstrated in renal tissue of patients with AAV (Figure 3) [138]. However, the risk of CVD in AAV is poorly quantified. A meta-data analysis of observational studies showed that there is an increased risk of CVD in patients with AAV [139]. However, NETs have been suggested to play a role in ANCA-associated thrombotic events, as thrombi obtained from ANCA vasculitis patients are rich in both NETs and histone citrullination [140,141]. NETs could play a dominant role in this relationship, but further research is required to reveal a causative correlation and more insight into mechanistic pathways.

Diabetes is a low-grade inflammatory autoimmune disease, and complications such as endothelial dysfunction, hyperreactivity of platelets and elevated levels of pro-coagulant mediators are common [142,143]. Neutrophils can infiltrate the pancreas in patients with type 1 diabetes (T1D) and play a role in the onset and the progression of T1D [144,145]. Studies have also demonstrated that neutrophils from T2D patients produced NETs at a greater rate than healthy controls [146]. Pancreas-residing neutrophils are capable of forming NETs [147], and studies evaluating neutrophil count in patients with T1D reveal a negative correlation between neutrophil counts and NE and proteinase 3 levels and activity increase, suggesting enhanced NETs formation [144,145,148,149]. The expression of PAD4 in neutrophils is increased in patients with both T1D and T2D compared to healthy controls, and in *PAD4^−/−^* mice NETs formation was reduced [150]. This highlights PAD4 as a potential therapeutic target to interfere with NETs formation. Clots from the citrated plasma of patients with diabetes mellitus showed greater prothrombotic effects than clots from the control [151]. Thus, NETs in diabetes could contribute greatly to the formation of thrombosis in diabetic patients.

A recent study in *Ldlr^−/−^* mice fed a WD for 16 weeks found that NETs+ plaques had upregulated NLRP3 inflammasome and glycolysis pathways, as observed by transcriptomic profiling [31]. This study also found that the occurrence of NETs declined in non-diabetic mice when hyperlipidemia was reduced followed by resolution of atherosclerosis. However, NETs persisted in diabetic mice under hyperglycemic conditions, exacerbating macrophage inflammation and impairing resolution. Diabetic mice treated with DNase 1 reduced plaque NETs content and macrophage inflammation, allowing for lesion regression. This suggests that NETs in diabetes can exacerbate atherosclerotic lesions, and DNase could be a potential therapeutic treatment in diabetes to reduce the risk of CVD. There is a strong correlation of T1D and T2D patients with CVD risk; one reason for this correlation is diabetic vascular complications induced by hyperglycemia, known to enhance ROS formation and lipotoxicity [152]. It could also be possible that the hyperglycemic conditions themselves trigger NETs formation spontaneously via the NADPH oxidase-dependent pathway [153], and increased ROS activity and lipotoxicity cause vascular damage [154,155]. A correlation between the occurrence of diabetes-induced organ damage, such as nephropathy or atherosclerosis, and cell-free DNA has been shown [156]. Additionally, it was shown that histone concentrations in plasma directly correlate with blood glucose levels [156]. However, a contrasting study has demonstrated that high glucose impairs NETs formation creating extracellular DNA lattices, which were short-lived and unstable, leading to rapid erosion [157]. The same study also showed that neutrophils induce NETs in an autocrine fashion by release of IL-6; however IL-6, failed to induce NETs in a high-glucose environment. Nevertheless, when glycolysis was inhibited in vitro, using 2-deoxyglucose (2-DG) it restored both LPS and IL-6 stimulated NETs formation in a high-glucose milieu. Yet, high-dose glucose and hyperglycemia were also shown to increase the release of NETs [156]. The differences in these results could be due to differing incubation times, as studies have demonstrated that acute glucose fluctuations, and not sustained chronic hyperglycemia, resulted in oxidative stress and were associated with the development of microvascular complications in T2D patients [158,159]. Thus, glycemic fluctuation in T2D may trigger NETs formation and cause or exacerbate CVD; however, further research is needed to investigate this hypothesis. Further research is needed to better understand the role of NETs in T2D, as in vitro results also showed that hyperglycemia could impair and delay NETs formation [157].

Psoriasis is a chronic, immune-mediated disease manifesting mainly as skin lesions and affects 2–3% of the world population [160,161]. Psoriasis is associated with systemic inflammation, similar to CVD, chronic obstructive pulmonary diseases and T2D mellitus [162]. One important mediator in psoriasis is lipocalin-2 (LCN2), which is an antimicrobial protein and adipokine associated with insulin resistance, obesity and atherosclerosis [163]. Serum LCN2 levels are elevated in psoriatic patients [163], and both granulocytes and keratinocytes, residing in the epidermis, secrete LCN2, driving the chemotaxis of neutrophils and sustaining NETs formation, thereby maintaining the psoriatic inflammation [164]. Regarding psoriasis, neutrophils release IL-17 in the process of forming NETs, which significantly contribute to IL-17-related endothelial dysfunction in both atherosclerosis and keratinocyte proliferation in psoriasis [165]. The latter could explain why atherosclerosis is associated with psoriasis [166]. Secukinumab, a monoclonal anti-IL-17 antibody, can clear the neutrophils in the epidermis and can improve psoriasis [167]. Moreover, secukinumab also had a beneficial effect on CVD risk by maintaining endothelial homeostasis in patients with psoriasis [168]. Beneficial effects of secukinumab in psoriasis patients may stem from the global clearance of neutrophils; however, the activity of NETs in psoriatic patients with CVD that are treated with secukinumab have not been investigated.

## 5. Conclusions

Neutrophils, as an essential component of the innate immune system, play a crucial role in the control of infectious diseases. In addition, they are involved in the pathogenesis of various inflammatory diseases. NETs formation may foster chronic inflammation, thereby promoting cardiovascular and autoimmune diseases, which in turn makes them an interesting therapeutic target. However, using therapeutic approaches such as DNAse I treatment to disrupt NETs remain controversial. Alternative lines to interfere or block NETs formation could be inhibition of PAD4, histone neutralization, ROS scavenging or partial clearance of neutrophils. Nevertheless, all the latter are still not sufficiently understood to reach a clinical state. Hence, a better understanding of NETs function and the balance of NETs induction versus inhibition is needed to move one step further into patient-tailored treatment approaches.

## Figures and Tables

**Table 1:** List of pathologies involved in the manifestation of NETs-induced vascular diseases.

Pathological Condition	NETs Inducers	Vascular Manifestation	References
Bacterial infection	Methicillin-resistant S. *aureus*, α-enolase, lipopolysaccharides, early secretory antigen-6 protein	Endocarditis, acute myocardial infection, myeloid cells recruitment to atherosclerotic lesions, microvascular thrombosis, thrombosis of injured heart valves, enhanced atherosclerotic lesion size	[7,11,12,13,14,15,16,17,18]
Viral infection	High levels of intracellular reactive oxygen species	Immunothrombosis, microvascular thrombi in the lung, kidney, and heart and organ damage	[19,20,21,22]
Atherosclerosis	Cholesterol crystal, interleukin (IL) IL-1β and IL-6, IL-8, oxLDL, high levels of anti-ApoA-1, metastasis-associated lung adenocarcinoma transcript 1 (MALAT1)	Endothelial damage in SMC-rich plaques, plaque macrophage inflammation, increased plaque vulnerability, enhanced lesion size and carotid artery thrombosis	[23,24,25,26,27,28,29,30,31,32]
Thrombosis	High mobility group box 1 (HMGB1), hemodynamic force	Occlusion of microvessels and bigger vessels, enhanced infarct size	[33,34,35,36,37]
Autoimmune diseases	low-density granulocytes (LDGs), neutrophil antimicrobial peptide LL37 and HNP	Proatherogenic NETs-derived lipoprotein oxidation, endothelial damage, macrophage inflammation and atherosclerosis	[31,38,39,40,41,42]

## Data Availability

Not applicable.
